# Revisiting bacterial volatile-mediated plant growth promotion: lessons from the past and objectives for the future

**DOI:** 10.1093/aob/mcy108

**Published:** 2018-07-05

**Authors:** Rouhallah Sharifi, Choong-Min Ryu

**Affiliations:** 1 Department of Plant Protection, College of Agriculture and Natural Resources, Razi University, Kermanshah, Iran; 2 Molecular Phytobacteriology Laboratory, Infectious Disease Research Center, KRIBB, Daejeon, South Korea; 3 Biosystem and Bioengineering Program, University of Science and Technology (UST), Daejeon, South Korea

**Keywords:** PGPR, bacterial volatile, BVC, biostimulant, field application

## Abstract

**Background:**

Bacterial volatile compounds (BVCs) are important mediators of beneficial plant–bacteria interactions. BVCs promote above-ground plant growth by stimulating photosynthesis and sugar accumulation and by modulating phytohormone signalling. These compounds also improve below-ground mineral uptake and modify root system architecture.

**Scope:**

We review advances in our understanding of the mode of action and practical applications of BVCs since the discovery of BVC-mediated plant growth promotion in 2003. We also discuss unanswered questions about the identity of plant receptors, the effectiveness of combination of two or more BVCs on plant growth, and the potential side effects of these compounds for human and animal health.

**Conclusion:**

BVCs have good potential for use as biostimulants and protectants to improve plant health. Further advances in the development of suitable technologies and preparing standards and guidelines will help in the application of BVCs in crop protection and health.

## INTRODUCTION

Volatile organic compounds (VOCs) are highly diffusible in soil and the plant canopy and play important roles in plant biology. Plants either take up some VOCs as nutrient sources or perceive them as infochemicals (Meldau *et al.*, 2013; Bailly *et al.*, 2014; Matsui, 2016; Aziz *et al.*, 2016; Zhou *et al.*, 2016). Plants are exposed to biogenic VOCs from various sources such as bacteria, fungi and other plants. Since 2003, when the first study was published on the promotion of plant growth by bacterial volatile compounds (hereafter referred to as BVCs), bacteria have been found to produce more than 1000 VOCs and non-organic compounds, such as HCN and NH_3_ (Audrain *et al.*, 2015). The main groups of BVCs are alkenes, ketones and alcohols (Penuelas *et al.*, 2014). Each bacterial strain releases a specific blend of BVCs, which play important roles in the bacterial life cycle and their interactions with other organisms including plants. For instance, some BVCs can regulate bacterial motility, antibiotic resistance and biofilm formation (Kim *et al.*, 2013), act as virulence-modulating factors for plant and animal pathogenic bacteria (Audrain *et al.*, 2015), and improve the growth and health of animals and plants (Ryu *et al.*, 2003; Bansal *et al.*, 2010; Sharifi and Ryu, 2016). Furthermore, each individual volatile does not necessarily play a single role that benefits the emitter organism (Huang *et al.*, 2012; Morath *et al.*, 2012; Bailly *et al.*, 2014). For example, bacterial indole influences antibiotic resistance (Audrain *et al.*, 2015), increases biofilm production in bacteria, stimulates plant growth (Bailly *et al.*, 2014), kills nematodes (Anyanful *et al.*, 2005) and has beneficial effects on the immunity of human intestinal epithelial cells (Bansal *et al.*, 2010).

In this review, we focus on the current understanding of the mechanism underlying plant growth promotion by BVCs obtained through intensive investigations using physiological and molecular tools, such as transcriptome and proteome analyses. We also provide insights into overcoming the limitations of BVCs for use in agriculture. We minimize our discussion of the effects of BVCs in elicitating plant systemic defence due to the availability of comprehensive reviews on this topic (Farag *et al.*, 2006; Audrain *et al.*, 2015; Ryu, 2015; Sharifi and Ryu, 2016). Based on a large number of studies, we summarize the positive effects of BVCs on plant growth (Blom *et al.*, 2011; Sánchez‐López *et al.*, 2016), from stimulating seed germination to enhancing fruit production (Table 1). BVCs increase above-ground plant cell size, leaf size and leaf number, enhance fruit yield and seed production, and increase below-ground lateral root and root hair formation, as well as nutrient uptake, photosynthetic activity and sugar accumulation. BVCs also regulate hormone signalling to improve plant growth and health (Ryu *et al.*, 2004; Sánchez‐López *et al.*, 2016; Tahir *et al.*, 2017). Therefore, these compounds have great potential for use in the field (Song and Ryu, 2013; Ryu, 2015). In this review, we provide answers to questions about BVC-mediated plant growth promotion, from its discovery to the underlying mechanism to field trials. We also discuss unanswered questions, which range from finding plant receptors for BVCs to using a mixture of BVCs for agricultural applications.

**Table 1.** Effects of rhizobacterial volatile organic compounds on plant morphology and physiology

Affected plant process	Plant species	Bacterial volatile compound or synthetic compound	Experimental condition	References
Shoot weight	*Arabidopsis*	2,3-Butanediol	I-plate	(Ryu *et al.*, 2003)
		Acetoin	Magenta box	(Rudrappa *et al.*, 2010)
	Alfalfa	Dimethylhexadecylamine	Petri dish	(Velázquez-Becerra *et al.*, 2011)
	Tomato	Albuterol and 1,3-propanediol	Pot assay	(Tahir *et al.*, 2017)
	Tobacco	*Pseudomonas fluorescens* SS101	Pot assay	(Park *et al.*, 2015)
Leaf surface area	*Nicotiana attenuata*	Dimethyl disulphide	Petri dish	(Meldau *et al.*, 2013)
	*Arabidopsis*	*Paenibacillus polymyxa* E681	Petri dish	(Lee *et al.*, 2012)
Cell size	*Arabidopsis*	*Bacillus subtilis* GB03	Petri dish	(Zhang *et al.*, 2007)
Chlorophyll content	Sorghum	Dimethyl hexadecylamine	Petri dish	(Castulo-Rubio *et al.*, 2015)
	*Arabidopsis*	*Bacillus subtilis* GB03		(Zhang *et al.*, 2008*b*)
	Soybean	*Pseudomonas simiae*	Magenta box	(Vaishnav *et al.*, 2015)
Flowering	*Arabidopsis*	*Bacillus subtilis* GB03	Magenta box	(Xie *et al.*, 2009)
Fruit production	Cucumber	3-Pentanol	Field	(Song and Ryu, 2013)
Seed production	*Arabidopsis*	*Bacillus subtilis* GB03	Magenta box	(Xie *et al.*, 2009)
Seed germination	Cabbage	Indole	I-plate	(Yu and Lee, 2013)
	*Codonopsis pilosula*	*Bacillus subtilis* GB03	I-plate	(Wu *et al.*, 2016)
Root proliferation	*Arabidopsis*	*Bacillus* sp.	I-plate	(Gutiérrez-Luna *et al.*, 2010)
		Indole	Vertical plate	(Bailly *et al.*, 2014)
		Indole	I-plate	(Bhattacharyya *et al.*, 2015)
Photosynthesis	Sorghum	Dimethylhexadecylamine	Glass flask	(Castulo-Rubio *et al.*, 2015)
	*Arabidopsis*	*Bacillus subtilis* GB03	I-plate	(Zhang *et al.*, 2008*b*)
Iron acquisition	*Arabidopsis*	*Bacillus subtilis* GB03	I-plate	(Zhang *et al.*, 2009)
	Sorghum	Dimethylhexadecylamine	Glass flask	(Castulo-Rubio *et al.*, 2015)
Sulphur acquisition	*Nicotiana attenuata*	Dimethyl disulphide	Petri dish	(Meldau *et al.*, 2013)
	*Arabidopsis*	*Bacillus subtilis* GB03	Magenta box	(Aziz *et al.*, 2016)
Sugar assimilation	*Arabidopsis*	*Bacillus subtilis* GB03	Petri dish	(Zhang *et al.*, 2008*b*)
Monoterpene synthesis	Peppermint	*Pseudomonas fluorescens*	I-plate	(Santoro *et al.*, 2011)
Auxin	*Arabidopsis*	Indole	I-plate	(Bhattacharyya *et al.*, 2015)
		Indole	Vertical plate	(Bailly *et al.*, 2014)
		*Bacillus subtilis* GB03	I-plate	(Zhang *et al.*, 2007)
	Tomato	*Bacillus subtilis SYST2*	Pot assay	(Tahir *et al.*, 2017)
Cytokinin	*Arabidopsis*	Indole	I-plate	(Bhattacharyya *et al.*, 2015)
		*Bacillus subtilis* GB03	I-plate	(Ryu *et al.*, 2003)
ABA	*Arabidopsis*	*Bacillus subtilis* GB03	I-plate	(Zhang *et al.*, 2008*b*)
Ethylene	*Arabidopsis*	*Bacillus subtilis* GB03	I-Plate	(Ryu *et al.*, 2003)
		*Bacillus subtilis* GB03	I-plate	(Kwon *et al.*, 2010; Lee *et al.*, 2012)
		*Paenibacillus polymyxa* E681	I-Plate	(Lee *et al.*, 2012)
	Tomato	*Bacillus subtilis SYST2*	Pot assay	(Tahir *et al.*, 2017)

## UPDATING BVC-ELICITED PLANT GROWTH PROMOTION

Since the discovery of BVC-induced growth promotion of *Arabidopsis thaliana* (*Arabidopsis*) in 2003, many studies have broadened our understanding of plant–bacteria interactions via volatile emissions. Here, we summarize previous questions and scientific trials aimed at obtaining complete answers on this topic.

### Bacterial volatiles promote plant growth

**Fig. 1.**
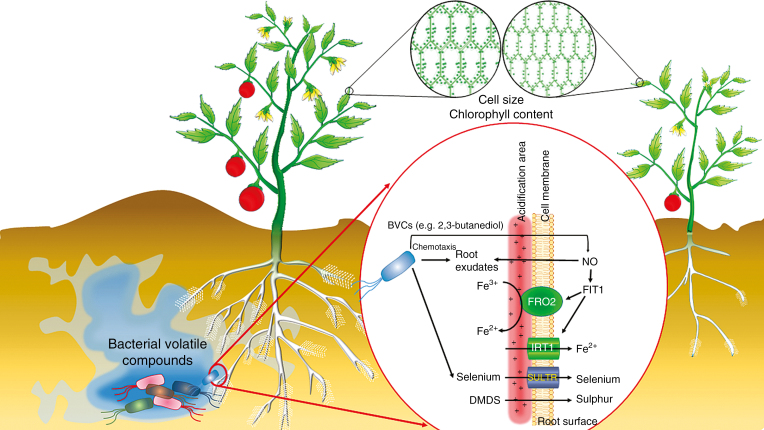
Bacterial volatiles improve plant growth and yield, leaf size, flower and fruit production, root proliferation, root hair formation, cell size, and chlorophyll content. Bacterial volatiles can help plants take up sulphur, selenium and iron. In the case of iron, volatiles enhance proton release to the rhizosphere and increase the expression of *FRO2* and *IRT1*, which are involved in the reduction and transport of iron, respectively. These genes are regulated by FIT1, expression of which is induced by nitric oxide (NO). Bacteria volatiles enhance NO accumulation in plants. Volatiles also increase selenium uptake by upregulating sulphate transporter genes (SULTRs). DMDS, dimethyl disulphide.

The effect of BVCs on plant growth was first discovered by Ryu *et al.* (2003), who found that treatment with volatiles from *Bacillus subtilis* GB03 increased plant growth in *Arabidopsis*. Analysis of volatile compound profiles suggested that 2,3-butanediol and its precursor acetoin are plant growth-promoting compounds (Farag *et al.*, 2006). Treatment of plants with 2 ng pure 2,3-butanediol in a 44.18-cm^3^ I-plate which divided two compartments in the Petri dish and analysis of a mutant bacterium lacking 2,3-butanediol biosynthesis gene(s) confirmed the importance of this compound to plant growth (Ryu *et al.*, 2003). The role of acetoin in the growth of *Arabidopsis* and tobacco was also confirmed by placing 1 mL of 10 mm acetoin in a 590-cm^3^ container (Xie *et al.*, 2009; Rudrappa *et al.*, 2010). Further studies uncovered the roles of specific volatiles or volatile blends at different stages of plant development. BVCs from some rhizobacteria enhance seed germination, increase leaf size and biomass production, induce early flowering, increase flower number, and improve fruit and seed production (Zhang *et al.*, 2007; Xie *et al.*, 2009; Song and Ryu, 2013) (Fig. 1). Various BVCs function during different steps in plant phenology. For example, *Proteus vulgaris* produces indole as its primary volatile, which increases the vigour index by up to 40 % in Chinese cabbage at an optimum concentration of only 0.63 ng per 44.18-cm^3^ I-plate (Yu and Lee, 2013). After seed germination, volatiles can also improve morphogenesis. *Bacillus badius* M12 volatiles induce morphogenesis in tobacco callus under tissue culture conditions and alleviate callus browning by inducing antioxidant biosynthesis (Gopinath *et al.*, 2015). Both bacterial and fungal VOCs increase plant cell size by modulating the expression of several genes involved in cell wall expansion and rigidity (Zhang *et al.*, 2007), including *Expansin* genes, which are important for cell wall extension (Zhang *et al.*, 2007; Minerdi *et al.*, 2011). Volatiles from *Bacillus megaterium* B55 increase leaf surface area and leaf number up to 4- and 2-fold, respectively, compared with the control (Meldau *et al.*, 2012). Similar findings were obtained for *Arabidopsis* treated with volatiles from *Paenibacillus polymyxa* E681 (Lee *et al.*, 2012). The combined effects of these compounds increase plant biomass up to 2.6- and 9.5-fold in *Arabidopsis* and tobacco, respectively (Park *et al.*, 2015; Tahir *et al.*, 2017).

Roots anchor plants in the soil and provide water and minerals for plant growth. Roots also provide a nutrient-rich environment for micro-organisms. Plant growth-promoting rhizobacteria (PGPR) volatiles, dimethylhexadecylamine (DMHDA) and indole, increase primary root length, lateral root length and number, and root hair density (Bailly *et al.*, 2014; Castulo-Rubio *et al.*, 2015). Consequently, these changes increase root volume and surface area (Fig. 1). However, some BVCs can suppress primary root growth while promoting lateral root growth and root hair formation. Volatiles from *Bacillus* spp. reduce meristem size in the root tip as well as reduce primary root length, whereas they significantly increase the number and length of lateral roots in an I-plate system (Gutiérrez-Luna *et al.*, 2010); the volatile indole is responsible for these changes (Bailly *et al.*, 2014). Treatment with 60–600 µg indole per 244.8 cm^3^ vertical plate conferred maximum lateral root volume and biomass production without negatively affecting primary root length, whereas 6000 µg indole had a similar effect on lateral root growth and significantly reduced primary root length (Bailly *et al.*, 2014). Bhattacharyya *et al.* (2015) demonstrated that 0.1 µg indole to a 44.18-cm^3^ I-plate is the optimal concentration for increasing lateral root number in *Arabidopsis*. A higher indole concentration of 0.63 µg indole per the same I-plate was optimum to increase Chinese cabbage root length (Yu and Lee, 2013). DMHDA from *Arthrobacter agilis* UMCV2 increases lateral root formation in sorghum at an optimum concentration of 8 µm in 10 µL of an ethanolic solution per 170 -mL flask (Castulo-Rubio *et al.*, 2015). This volatile is also effective for increasing root proliferation in alfalfa, with an optimum concentration of 32 µm (Velázquez-Becerra *et al.*, 2011). Treatment with only 0.5 µL (500 µg) of the volatile dimethyl disulphide (DMDS) per 44.18-cm^3^ I-plate doubled lateral root formation in *Nicotiana attenuata* (Meldau *et al.*, 2013). The increase in root volume expands the rhizosphere volume. The higher microbe population size and lower pH of the rhizosphere improve the availability of nutrients, especially phosphorus and iron, in calcareous soils (Pii *et al*., 2015; Sharifi and Ryu, 2017). Therefore, expanding the rhizosphere volume appears to be a successful strategy for both plants and rhizosphere bacteria (rhizobacteria).

Information about BVC blend and BVC-mediated plant–bacteria interactions mainly comes from I-plate systems (Table 1). The *in vitro* optimum concentration of some discrete volatiles on plant growth has previously been given. It is known that *Enterobacter aerogenes* released 1 µg/3 h of 2,3-butanediol in maize phyllosphere under glasshouse conditions (D’Alessandro *et al.*, 2014), and gut bacteria produce 250–1100 µm indole in the human intestine (Bansal *et al.*, 2010). However, we do not know the actual concentration of BVCs in the rhizosphere. However, plants are known to respond to low concentrations of volatiles such as 2,3-butanediol and indole (Ryu *et al.*, 2003; Bailly *et al.*, 2014; Bhattacharyya *et al.*, 2015).

### BVCs improve the yield and quality of crop plants

Treatment with BVCs can improve crop quality and yields, including seed, fruit, tuber, biomass, essential oil, secondary metabolite and sugar yields. *Bacillus subtilis* GB03 volatiles and benzaldehyde increase biomass and essential oil contents in medicinal plants *Codonopsis pilosula* and *Atractylodes lancea*, respectively (Wu *et al.*, 2016; Zhou *et al.*, 2016). The accumulation of sugars such as glucose, sucrose and starch is necessary for the quality of crops such as potato and sugar beet. Volatiles from several bacteria and fungi can increase the accumulation of these sugars (Zhang *et al.*, 2008*b*; Ezquer *et al.*, 2010; Sánchez‐López *et al.*, 2016). Some BVCs do not significantly increase plant biomass, but they do induce flowering and fruit production. For example, 3-pentanol and 2-pentanone had no effect on cucumber biomass but increased fruit production approximately 6- and 4-fold in the field, respectively (Song and Ryu, 2013). Treatment with volatiles from *B. subtilis* GB03 and from *Alternaria alternata* also increase flowering time, silique number and seed production in *Arabidopsis* under laboratory conditions (Xie *et al.*, 2009; Sánchez‐López *et al.*, 2016).

### Mode of action 1: BVCs modulate plant photosynthesis

BVCs can improve key steps in plant physiology, such as photosynthesis and carbohydrate accumulation, by increasing chlorophyll content and photosynthetic efficiency. BVCs increase chlorophyll content in *Arabidopsis* (Zhang *et al.*, 2009) and sorghum (Castulo-Rubio *et al.*, 2015). The effects of BVCs on chlorophyll content and photosynthesis occur via two mechanisms. The first involves iron, which is necessary for chlorophyll biosynthesis, electron transport chain activity and photosystem activity in plants (Briat, 2007). Rhizosphere acidification improves iron solubility and facilitates iron uptake. The soluble Fe^3+^ is then reduced to Fe^2+^ by FERRIC REDUCTASE OXIDASE 2 (FRO2) and transferred to the cytosol by IRON-REGULATED TRANSPORTER 1 (IRT1) (Lemanceau *et al.*, 2009). Zhang *et al.* (2009) showed that *B. subtilis* GB03 volatiles improve all of these steps in *Arabidopsis* iron uptake in an I-plate system (Fig. 1). Volatiles increase proton release by up to 3-fold, consequently acidifying the rhizosphere, and thus favouring iron solubility. Volatiles also upregulate the expression of FE-DEFICIENCY-INDUCING TRANSCRIPTION FACTOR1 (*FIT1*), increasing the expression of *FRO2* by up to 4-fold and that of *IRT1* by 10−20-fold. The iron content of volatile-treated plants doubled under this treatment. Similar results were obtained by Wang *et al.* (2017), who showed that *Bacillus amyloliquefaciens* BF06 volatiles increase nitric oxide (NO) accumulation in *Arabidopsis*. NO not only chelates and mobilizes iron, but it also acts upstream of the transcription factor FIT1 in plants under Fe-deficiency conditions (Fig. 1). These results indicate that NO plays a critical role in BVC-mediated iron uptake.

The second mechanism concerns alleviation of the negative feedback of sugar accumulation on photosynthesis by BVCs (Zhang *et al.*, 2008*b*). *Arabidopsis* HEXOSE SENSOR KINASE 1 (HXK1) senses hexose sugar concentrations after their accumulation during photosynthesis. HXK1 negatively regulates photosynthetic reactions by sensing high concentrations of sugars (Cho *et al.*, 2010). Plants treated with *B. subtilis* GB03 accumulated 60 % more hexose sugars than control plants (Zhang *et al.*, 2008*b*). Gene expression studies showed that BVCs repress HXK1 signalling in *Arabidopsis*, which was also observed using other microbial volatiles. Volatiles from *A. alternata* enhance plant growth (~4-fold) and improve photosynthesis efficiency parameters in a manner similar to volatiles from *B. subtilis* GB03 (Sánchez‐López *et al.*, 2016), and they increase glucose, fructose, sucrose and starch accumulation, 3-, 3.5-, 2- and 10-fold, respectively (Sánchez‐López *et al.*, 2016).

### Mode of action 2: increasing mineral uptake

PGPR facilitate the uptake of macro- and microelements in plants (Zhang *et al.*, 2009; Meldau *et al.*, 2013; Aziz *et al.*, 2016). BVCs from some PGPR strains improve the uptake of iron, copper, selenium and sulphur (Fig. 1). *Arthrobacter agilis* UMCV2 volatiles improve iron acquisition in both monocot and dicot plants (Castulo-Rubio *et al.*, 2015). Treatment with *B. subtilis* GB03 increased iron uptake up to 2-fold in *Arabidopsis*, even under alkaline conditions (Zhang *et al.*, 2009; Wang *et al.*, 2017). *Bacillus amyloliquefaciens* BF06 volatiles increase selenium uptake by inducing the expression of sulphate transporter genes in this plant: the selenium content was 23 % higher in volatile-treated plants than in untreated plants (Wang *et al.*, 2017). Furthermore, some BVCs can be consumed as a source of nutrients. DMDS, as a source of sulphur, increases *Arabidopsis* growth in sulphur-deficient medium (Meldau *et al.*, 2013) and *Atractylodes lancea* may uptake nitrogenous volatiles as a source of nitrogen (Zhou *et al.*, 2016). Together, these findings indicate that BVCs can increase nutrient uptake and transport in plants. These processes should be taken into consideration when developing strategies for improving fertilizer uptake efficiency using an integrated nutrient management approach.

### Mode of action 3: alleviating biotic and abiotic stress

BVCs indirectly improve plant growth by alleviating biotic and abiotic stress. Some BVCs, such as DMDS and 2-methylpentanoate, are highly toxic to plant pathogens (Groenhagen *et al.*, 2013; Cordovez *et al.*, 2015; Raza *et al.*, 2016; Ossowicki *et al.*, 2017), and some, such as acetoin, 2,3-butanediol and tridecane, induce plant systemic resistance (ISR) against these pathogens (Lee *et al.*, 2012). However, ISR appears to be the main mechanism of disease suppression via BVCs under natural conditions (Sharifi and Ryu, 2016). Some BVCs can also induce systemic tolerance to soil salinization and drought stress, which pose major threats to crop production. Treatment with rhizobacteria can help alleviate these problems by improving root system architecture for more efficient water uptake. Rhizobacteria confer systemic tolerance to abiotic stress by modulating proline, antioxidant and hormone production and reducing Na^+^ accumulation in plants (Liu and Zhang, 2015; Ngumbi and Kloepper, 2016; Sharifi and Ryu, 2017). BVCs from *B. subtilis* GB03 promote basipetal movement of Na^+^ from shoot to root by modulating the activity of HKT1, an *Arabidopsis* Na^+^ transporter (Zhang *et al.*, 2008*a*). In addition, treatment with GB03 BVCs increases choline and glycine betaine biosynthesis in *Arabidopsis* 2- and 5-fold, respectively (Zhang *et al.*, 2010). The volatile 2,3-butanediol helps protect plants from abiotic stress. Treatment with the *Pseudomonas chlororaphis* O6 mutant, which cannot synthesize 2,3-butanediol, failed to increase drought stress tolerance in *Arabidopsis* compared with the wild type. The plant hormones salicylic acid (Cho *et al.*, 2008) and NO (Cho *et al.*, 2013) are required for the plant response to 2,3-butanediol under abiotic stress.

### Mode of action 4: modulating hormone cross-talk

Some BVCs regulate plant growth by modulating the biosynthesis, perception and homeostasis of the plant hormones ethylene, auxin, cytokinin, abscisic acid (ABA) and gibberellin (Table 1). The *Arabidopsis ethylene insensitive 2* (*ein2*) mutant is less responsive to *B. subtilis* GB03 volatiles compared with the wild type (Ryu *et al.*, 2003), as mentioned in the first report about the role of bacterial volatiles in modulating phytohormone responses. BVCs from *P. polymyxa* E681 also failed to increase growth in *ein2* mutants, but they were effective in salicylic acid, jasmonic acid and gibberellin mutants (Lee *et al.*, 2012).

Rhizosphere bacteria also promote plant growth by stimulating auxin production or by modulating auxin homeostasis (Ruzzi and Aroca, 2015). Plants treated with *B. subtilis* GB03 BVCs display enhanced root proliferation via increasing lateral root formation through the auxin-dependent pathway (Zhang *et al.*, 2007). The volatile indole also modulates auxin signalling in *Arabidopsis* (Bailly *et al.*, 2014; Bhattacharyya *et al.*, 2015). Plants can take up indole and use it as a precursor for auxin production. Indeed, ^13^C indole was taken up by *Arabidopsis* and transformed into auxin through the tryptophan pathway (Bailly *et al.*, 2014). In addition, treatment with indole from *P. vulgaris* enhanced *Arabidopsis* seedling growth by up to 50 %, whereas auxin mutants and N-1-naphthylphthalamic acid-treated *Arabidopsis* plants did not respond to this volatile compound (Bhattacharyya *et al.*, 2015).

The role of BVCs in cytokinin signalling has been demonstrated in several studies. This role is important because cytokinin signalling can increase photosynthesis and flower production (Werner *et al.*, 2001). Ryu *et al.* (2003) reported that the response to volatiles from *B. subtilis* GB03 is impaired in the *Arabidopsis cytokinin receptor-deficient 1* (*cre1*) mutant. *Proteus vulgaris* and its volatile indole also failed to promote growth in this *cre1* mutant (Bhattacharyya *et al.*, 2015). Volatiles from *A. alternata* increase cytokinin accumulation (3-fold) in *Arabidopsis* (Sánchez‐López *et al.*, 2016), and they increase photosynthesis and reduce the time of floral bud appearance (3 d) through cytokinin signalling *in vitro*. Although fungi and bacteria produce different volatile profiles, transcriptome analysis showed that the plant response to *A. alternata* is quite similar to the response to *B. subtilis* GB03. Approximately 25 % of these differentially regulated genes are cytokinin-responsive (Sánchez‐López *et al.*, 2016). These findings indicate that plants respond to VOCs through a highly conserved signalling network.

ABA biosynthesis occurs when sugar accumulates as an end product of photosynthesis (Sánchez‐López *et al.*, 2016). ABA inhibits the accumulation of additional sugar by negatively affecting photosynthesis (Rolland *et al.*, 2006; Cho *et al.*, 2010). However, *B. subtilis* GB03 BVCs bypass this negative feedback by reducing ABA biosynthesis (Zhang *et al.*, 2008*b*). ABA concentrations in aerial plant parts were 50 % lower in plants treated with *B. subtilis* GB03 volatiles than in untreated plants. Treatment with *B. subtilis* GB03 failed to increase photosynthetic efficiency in plants treated with exogenous ABA.

## UNANSWERED QUESTIONS

Although the effects of BVCs on plant growth were discovered 15 years ago, the details of this phenomenon in terms of plant morphology, physiology and hormonal signalling have only recently been described (Table 1). Of the many unanswered questions, we will discuss four critical ones that remain to be answered.

### Can we identify the plant receptors for BVCs?

**Fig. 2.**
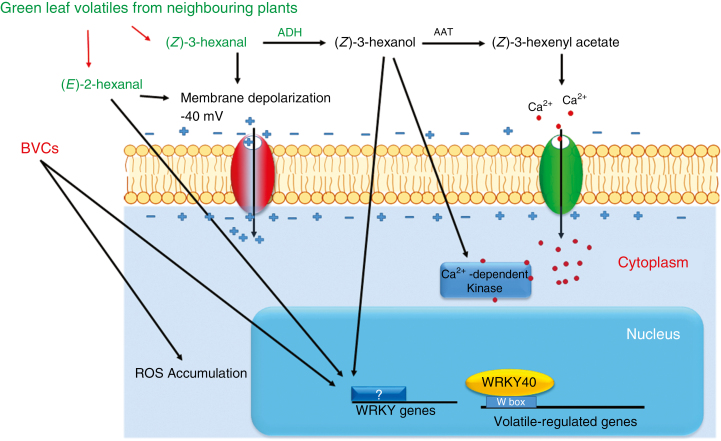
Volatile perception and signalling in plants. Herbivore-wounded plants release volatiles such as (*z*)-3-hexenal and (*E*)-2-hexenal, which deter herbivores from attacking the wounded leaves and inform neighbouring plants of the attack. These compounds elicit changes in plasma membrane potential depolarization and activate several regulatory proteins such as WRKY transcription factors. These volatiles are also converted to more active, highly volatile compounds such as (*Z*)-3-hexenol and (*Z*)-3-hexenyl acetate, which induce calcium influx and the expression of several regulatory genes, such as calcium-dependent kinase and WRKY genes. Bacteria volatiles induce the expression of *WRKY18* and ROS accumulation in plants.

The olfactory system was first identified as the site of volatile perception in animals in 1991 (Buck and Axel, 1991). Buck and Axel won the Nobel Prize in Physiology or Medicine for their outstanding study leading to the discovery of this perception system (Miller, 2004). However, the molecular mechanisms involved in plant volatile perception are still being elucidated. Most of our current knowledge about this topic was derived from studies on plant perception of C6 green leaf volatiles (GLVs). We can use this information to obtain hints about plant receptors of BVCs (Fig. 2). GLVs are produced in leaves damaged by herbivores. These compounds are involved in the interactions of plants with other plants and animals, as well as microbes (Scala *et al.*, 2013). GLVs such as (*z*)-3-hexenal, (*E*)-2-hexenal and (*z*)-3-hexenyl acetate accumulate in plants after herbivore attack (Zebelo *et al.*, 2012). These volatiles diffuse into the air to reach neighbouring plants. These compounds induce the depolarization of plasma membrane potential within a few seconds after treatment. (*E*)-2-hexenal induces the generation of reactive oxygen species (ROS) in *Arabidopsis* leaves, followed by transient Ca^2+^ influx just 3 min after exposure (Asai *et al.*, 2009). Treatment with (*z*)-3-hexenyl acetate also significantly increases Ca^2+^ influx into the cytosol less than 30 min after exposure (Zebelo *et al.*, 2012). Transcriptome analysis showed that genes encoding several Ca^2+^-dependent proteins, such as calmodulin-dependent protein kinase, and several proteins involved in lipid signalling, such as AOS and Lox5, were upregulated after treatment with the alcohol (*z*)-3-hexenol (Engelberth *et al.*, 2013). Some bacterial (Wenke *et al.*, 2012; Cho *et al.*, 2013) and fungal (Splivallo *et al.*, 2007) volatiles also induce ROS accumulation in plants, but this occurs more than 24 h after treatment. Two distinct peaks of ROS production occur after plant stress. The first phase, which is rapid and transient, induces downstream signalling pathways such as mitogen-activated protein kinase (MAPK) cascades (Mittler *et al.*, 2011). The second phase involves massive and prolonged ROS production, which functions in the hypersensitive reaction and the inhibition of microbes. Future work should focus on the role of BVCs in early ROS and Ca^2+^ signalling.

What is the downstream signalling pathway that functions after volatile perception? The W-box motif (TTGACY) is a common *cis*-regulatory element in genes that are upregulated 3 h after treatment with (*E*)-2-hexenal (Mirabella *et al.*, 2015). This motif is the binding site for WRKY transcription factors. Treatment with (*E*)-2-hexenal increases the expression of *WRKY40*, indicating that WRKY transcription factors act downstream of volatile perception (Mirabella *et al.*, 2015). Some WRKY transcription factors such as WRKY7 have a calmodulin-binding site and act downstream of Ca^2+^ signalling (Park *et al.*, 2005). However, the relationship between Ca^2+^ and WRKYs in volatile perception and signalling remains to be further elucidated. Volatiles from the plant growth-inhibiting bacteria *Serratia plymuthica* and *Stenotrophomonas maltophilia* also activate the transcription of genes for several W-box-enriched transcription factors in receiver plants, such as WRKY18 (Wenke *et al.*, 2012). These findings indicate that, at least during some steps, conserved regulatory systems respond to GLVs and BVCs.

Aldehydes are highly toxic to plant cells and act as anti-feeding signals for herbivores (Sugimoto *et al.*, 2014). In neighbour plants or leaves, these compounds are converted to alcohols or glycosides, which are less toxic, thereby having greater potential to function as signals. For example, (*z*)-3-hexenal can be converted to (*z*)-3-hexenol or (*z*)-3-hexenyl acetate, which are less toxic and more volatile than (*z*)-3-hexenal (Matsui *et al.*, 2012) and strongly affect Ca^2+^ influx (Engelberth *et al.*, 2013). The same phenomenon was also reported for BVCs. Some plants can bio-transform the volatile acetoin to different isoforms of 2,3-butanediol (Javidnia *et al.*, 2016). However, both compounds are active in plant growth promotion. Further research is needed to characterize the mechanisms of volatile perception and downstream signalling pathways in plants, especially those that function in the perception of BVCs (Fig. 2). There is a well-known trade-off between growth and defence, indicating that the activation of one process may have a negative effect on the other (Lozano-Duran and Zipfel, 2015; Campos *et al.*, 2016). To investigate the use of volatiles to improve plant growth, both plant growth promotion and induced resistance could simultaneously be recorded to help determine how single volatiles such as 2,3-butanediol prime strong defence responses (Ryu *et al.*, 2004) and improve plant growth approx. 4-fold (Ryu *et al.*, 2003). These questions could be answered by identifying the signalling pathway downstream of volatile perception.

### Are BVC mixtures more effective than single?

Bacteria produce blends of compounds in their volatile profiles (Farag *et al.*, 2006). The ratio and concentration of each volatile vary under different conditions (Blom *et al.*, 2011). We have described the beneficial properties of a few individual volatiles for plants, such as 2,3-butanediol, acetoin, indole, DMDS, DMHDA and 3-pentanol. The effective concentration has been defined for each of these compounds. According to our literature review, there are no reports on the effects of applying mixtures of bacterial volatiles for plant growth. However, volatile mixtures have excellent potential to further improve plant health, and the use of mixtures might optimize any potential positive effects of these compounds. Each VOC has a specific mode of action on plant growth (Table 1), and a combination of volatiles with different modes of action might have synergistic effects on plant growth. It is possible to mix bacterial volatiles to achieve both growth and ISR to pests and diseases. Indole appears to be a good candidate compound for agricultural application, as it increases plant biomass and root volume (Bailly *et al.*, 2014) and attracts natural enemies of pests (Erb *et al.*, 2015). The combined use of indole with effective volatiles against plant pathogens, such as acetoin, could improve the efficiency of the mixture. It might be possible to mix PGPR volatiles with agrochemicals. A mixture of benzothiadiazole (BTH) and 3-pentanol could be effective against several plant pathogens (Choi *et al.*, 2014). The compatibility of chemical ingredients in these mixes should be confirmed before use. However, organisms are evolutionarily attuned to relative concentrations of volatiles than to absolute amounts.

### Can BVCs trigger indirect defence against insect pests?

Bacterial volatiles can improve plant growth and defence responses by modulating physiological pathways. Can bacterial volatiles modulate plant volatile biosynthetic pathways? If so, they could help plants recruit natural enemies for the biological control of herbivore pests, thereby promoting plant growth. Indeed, one report indicates that BVC treatment can alter the essential oil content in dry leaves (Zhou *et al.*, 2016). Jasmonic acid is a central regulator of herbivore-induced plant volatile biosynthesis, and bacterial volatiles can regulate the jasmonic acid biosynthesis and signalling pathway (Pineda *et al.*, 2013; Sharifi and Ryu, 2016; Sharifi *et al.*, 2018). Therefore, perhaps BVCs could regulate plant volatile content. Indeed, there are several examples of plant-associated bacteria and fungi altering the volatile composition of living plants (Sharifi *et al.*, 2018). Mycorrhizae were shown to alter the volatile composition of common bean (Schausberger *et al.*, 2012). The treated plants synthesized β-caryophyllene and β-ocimene *de novo*. These compounds attract parasitoids of spider mites to treated plants (Schausberger *et al.*, 2012). Pineda *et al.* (2013) reported that treatment with the root-associated bacterium *Pseudomonas simiae* WCS417r altered the volatile composition of *Arabidopsis* via the jasmonic acid signalling pathway. However, this change in volatile composition had negative effects on the performance of parasitoids and the sucking insect *Myzus persicae*. By contrast, treatment with *P. simiae* WCS417r increased the attraction of parasitoids to the chewing insect *Mamestra brassicae* (Pangesti *et al.*, 2015). Root-associated bacteria treatment suppressed methyl salicylate and (*E*)-α-bergamotene biosynthesis in inoculated plants. Colonization of aerial tissues also altered root volatile emissions in plants. Leaf colonization by the endophytic fungus *Neotyphodium uncinatum* reduced the concentrations of plant volatiles such as monoterpenes but increased CO_2_ emissions (Rostás *et al.*, 2015). Treatment of cucumber plants with the volatiles 3-pentanol and 2-butanone in the field increased the number of ladybird beetles, a natural enemy of aphid pests (Song and Ryu, 2013). These volatiles induce jasmonic acid signalling, a key modulator of plant volatile emissions. However, in this study, the volatile profiles in treated and non-treated plants were not analysed. Future work is needed to investigate the effects of bacteria volatiles on the emission of plant volatiles that indirectly contribute to plant defence.

### Do BVCs have any side effects for animal and human health?

We described several pure volatiles as plant growth activators. However, we should consider that volatiles are a double-edged sword. Some play multiple roles for emitter micro-organisms, and some of these roles have negative effects on non-target organisms and human health. For example, DMDS enhances root proliferation and sulphur uptake in plants (Meldau *et al.*, 2013), but it also has insecticidal activity by inhibiting electron transfer in insects (Gautier *et al.*, 2008). DMDS also has negative effects on nematodes and *Drosophila melanogaster* (Popova *et al.*, 2014). The lethal concentration 50 (LC_50_) of DMDS in rat is 4.1 ppm, which is considerably lower than that of most pesticides (Korpi *et al.*, 2009). Inhalation of this compound by humans can produce headaches and loss of vigour (Korpi *et al.*, 2009). The volatile 1-octen-3-ol is a good candidate for use as an activator of plant health and an inducer of resistance to plant pathogens (Kishimoto *et al.*, 2007); however, it negatively affects the nervous and respiratory systems of *D. melanogaster* (Bennett and Inamdar, 2015) and irritates the eyes and respiratory system in humans (Araki *et al.*, 2010). This volatile had cytotoxic activity on human lung cell lines in cell culture (Korpi *et al.*, 2009). It should be mentioned that high concentrations of 1-octen-3-ol induce an oxidative burst in *Arabidopsis* (Spilvallo *et al.*, 2007). BVCs can have negative effects on human health via indirect pathways. These compounds can increase resistance to antibiotics or enhance the virulence of human-associated pathogenic bacteria. Volatiles from *Burkholderia ambifaria* confer resistance to kanamycin and gentamicin in *Escherichia coli* (Groenhagen *et al.*, 2013). The volatiles trimethylamine and ammonia from Gram-negative bacteria increase resistance to tetracycline at long distances (Létoffé *et al.*, 2014). Volatiles also affect bacterial motility, antibiotic resistance and biofilm formation. The plant growth-promoting volatiles indole and acetoin modulate motility and biofilm formation in various human-associated bacteria (Kim *et al.*, 2013; Létoffé *et al.*, 2014). Therefore, we should consider that volatiles are highly active and that they can have negative or unknown effects on non (off)-target organisms, including humans. Robust safety regulations will be needed for the commercial release of BVCs, such as those required for chemical pesticides. Safety information should be provided by authorized laboratories. This is very important for farmers when applying BVCs in the field.

## PERSPECTIVES

BVCs are the ‘chemical language’ that bacteria use to interact with their plant partners. These compounds modulate plant physiological and hormonal pathways to increase biomass and yield production. BVC-treated plants exhibit increased root volume, leaf number, leaf size and flower number, allowing for higher fruit and seed production. These features indicate that BVCs might be used as fertilizers in bio-farming. More than 1000 BVCs have been identified to date, but only a dozen of these have been characterized in detail. Further studies are needed to identify more effective volatiles and to determine their effective concentrations, as well as to investigate the effects of artificial volatile mixtures on plant growth under glasshouse and field conditions. However, we must consider the side effects of these volatiles, which are highly active and potentially hazardous. Some volatiles that are effective for use in plants have adverse side effects on non-target organisms such as insects, nematodes and humans. Therefore, extensive testing will be required prior to the commercial release of these compounds. In this review, we have attempted to update the recent information and address unanswered questions on BVC research to guide future studies aimed at addressing gaps in our knowledge.
